# Pediatric emergency medical care in Yerevan, Armenia: a knowledge and attitudes survey of out-of-hospital emergency physicians

**DOI:** 10.1186/1865-1380-7-11

**Published:** 2014-02-07

**Authors:** Aline A Baghdassarian, Ross I Donaldson, Andrew D DePiero, Nancy L Chernett, Harsh Sule

**Affiliations:** 1 Division of Emergency Medicine, Nemours/Alfred I. duPont Hospital for Children, 1600 Rockland Rd, Wilmington, Delaware 19803, USA; 2 Department of Emergency Medicine, Thomas Jefferson University Hospital, 1020 Sansom Street, Philadelphia, PA 19107, USA; 3 Jefferson School of Population Health, Thomas Jefferson University, 901 Walnut Street, 10th Floor, Philadelphia, PA 19107, USA; 4 Jefferson Medical College, Thomas Jefferson University, 1025 Walnut Street, Philadelphia, PA 19107, USA; 5 Department of Emergency Medicine, Harbor-UCLA Medical Center, 924 Westwood Boulevard, Suite 300, Los Angeles, CA 90095, USA; 6 UCLA School of Public Health, 640 Charles EYoung Dr S, Los Angeles, CA 90024, USA; 7 Division of Pediatric Emergency Medicine, Virginia Commonwealth University Medical Center Main Hospital, 2nd Floor, Ste 2-500, 1250 E. Marshall Street, P.O. Box 980401, Richmond, VA 23298-0401, USA

**Keywords:** Armenia, Yerevan, Emergency medical services development, Emergency medical services for children, International pediatric emergency medicine, Pediatric emergency care education, Pediatric emergency medicine

## Abstract

**Background:**

Out-of-hospital emergency care is at an early stage of development in Armenia, with the current emergency medical services (EMS) system having emergency physicians (EPs) work on ambulances along with nurses. While efforts are underway by the Ministry of Health and other organizations to reform the EMS system, little data exists on the status of pediatric emergency care (PEC) in the country. We designed this study to evaluate the knowledge and attitudes of out-of-hospital emergency physicians in pediatric rapid assessment and resuscitation, and identify areas for PEC improvement.

**Methods:**

We distributed an anonymous, self-administered Knowledge and Attitudes survey to a convenience sample of out-of-hospital EPs in the capital, Yerevan, from August to September 2012.

**Results:**

With a response rate of 80%, the majority (89.7%) of respondents failed a 10-question knowledge test (with a pre-defined passing score of ≥7) with a mean score of 4.17 ± 1.99 SD. Answers regarding the relationship between pediatric cardiac arrest and respiratory issues, compression-to-ventilation ratio in neonates, definition of hypotension, and recognition of shock were most frequently incorrect. None of the participants had attended pediatric-specific continuing medical education (CME) activities within the preceding 5 years. χ^2^ analysis demonstrated no statistically significant association between physician age, length of EMS experience, type of ambulance (general vs. resuscitation/critical care), or CME attendance and pass/fail status. The majority of participants agreed that PEC education in Armenia needs improvement (98%), that there is a need for pediatric-specific CME (98%), and that national out-of-hospital PEC guidelines would increase PEC safety, efficiency, and effectiveness (96%).

**Conclusions:**

Out-of-hospital emergency physicians in Yerevan, Armenia are deficient in pediatric-specific emergency assessment and resuscitation knowledge and training, but express a clear desire for improvement. There is a need to support additional PEC training and CME within the EMS system in Armenia.

## Background

The emergency medical services (EMS) system in Armenia is based on the “Franco-German” model of emergency medical care wherein physicians work on ambulances along with nurses. Consistent with this model, Armenian emergency physicians (EPs) provide patient care at the scene and have a very low transfer rate to hospital (<15%) [1]. Therefore, the term “out-of-hospital” is used instead of “pre-hospital” when describing these EPs. In Armenia, out-of-hospital EPs are not part of a separate specialty; instead, they are often trained in other specialties or begin work immediately after completing a post-medical school internship year. They generally do not have pediatric-specific training and there are no nationally accepted or widely used EMS guidelines or protocols to guide the care of pediatric patients in the country. This is significantly different compared to the “Anglo-American” or “Specialty” model of emergency medicine whereby pre-hospital care is delivered by non-physicians with the goal of rapid transport to a hospital-based emergency department where a specifically trained EP delivers patient care. Additionally, in the absence of a medical command system, physicians who are inadequately trained in pediatric emergency care (PEC) have limited options to seek supervision when responding to these cases.

The need to improve the Armenian EMS system has been recognized by both the medical community and government officials since independence from the Soviet Union in 1991. From 1993 to 1997, a partnership between the United States Agency for International Development (USAID), the University of Massachusetts, and the Ministry of Health (MoH) of Armenia resulted in the development of a regional training center. Here, over 1,800 health care workers and first responders were trained with a mean improvement in test scores of 100% in ambulance drivers and police, fire, and military personnel, and 58–60% in physicians and nurses [2]. However, while the training center continues to function, few additional updates have been made to the EMS system or provider education in Armenia.

The MoH of Armenia is currently working to modernize the EMS system. However, there is a significant gap in information about the current state and while it would be possible to simply invest money in new equipment, successful development experience indicates that building human capacity is critical to success. Therefore, we designed a study to evaluate the human capacity for PEC via a knowledge and attitudes survey of pediatric rapid assessment and resuscitation in out-of-hospital EPs. To our knowledge, this is the first study of its kind in Armenia. The goal of this study is to identify areas of improvement regarding PEC education and the pediatric EMS system in Yerevan, Armenia.

## Methods

### Study setting

Yerevan is the capital of Armenia, with a population of 1.1 million and encompassing 87.65 sq. miles [3,4]. It has one central dispatch center and 6 sub-centers and 35 ambulances (10 resuscitation/critical care ambulances and 25 general/basic ambulances), with a total staff of 650, of which 200 are physicians. A nurse or physician at the dispatch center receives calls made to the 1-03 emergency ambulance number.

**Figure 1:**
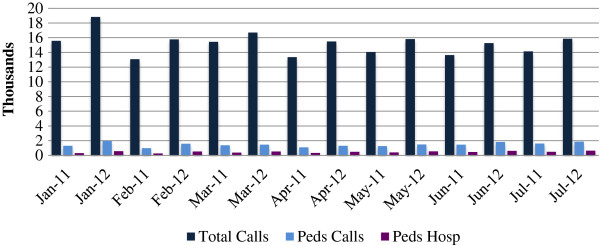
**EMS utilization 2011–2012: proportion of pediatric EMS calls and pediatric hospitalizations 2011 and 2012 – Central Dispatch Center Surveillance Data **[5].

According to surveillance data from the Yerevan Central Dispatch Center, EMS utilization has increased over the past two years: total EMS calls were 12% higher in July 2012 compared to July 2011, while pediatric EMS calls were 15% higher. Pediatric calls constituted 10–12% of all EMS calls in both 2011 and 2012 (Figure 1) [5].

### Study design

This was a cross-sectional, anonymous, self-administered survey study. The knowledge questions were designed based on PEC knowledge taught in standard PEC courses and textbooks. These questions were then further validated via pilot testing with groups of pediatric emergency physicians in the US and a group of physicians in Yerevan.

The questions were drafted in English and then translated into Eastern Armenian, the official language of Armenia. The translation was reviewed by the lead author who is a native Armenian speaker, by physicians in Yerevan who are not part of this study and are fluent in both English and Armenian and by native Armenian-speaking faculty at Columbia University, New York, who are also fluent in English. The survey was back translated into English and it was verified that there was no change in the substance or form of the questions.

The study was given exempt status by the Institutional Review Board at Thomas Jefferson University and approved by the MoH of Armenia.

### Data collection

From August to September 2012, we distributed the questionnaires to a convenience sample of physicians working in the Yerevan EMS system (n = 170). Participants returned the completed surveys to the Central Dispatch Center, where they were picked up at the end of the study period.

### Data analysis

We utilized the Statistical Package for Social Science (SPSS**®**, Version 17.0) for data analysis and first performed a descriptive analysis of distributions, means, medians, and proportions to characterize demographics of the study participants. Based on a pre-defined protocol and the 10 individual knowledge questions, we then calculated a Knowledge Score (number correct per respondent) and a dichotomous pass/fail status for each respondent. Further analysis correlated mean Knowledge Scores with different age groups, length of EMS work experience, pediatric-specific training, and continuing medical education (CME) sessions attended, using a statistical significance of *P* ≤0.05. Physician attitude items utilized 4-point Likert scales, with responses collapsed into dichotomous variables followed by frequency analysis to report participant responses. We included surveys with incomplete responses in the analysis.

## Results

The survey response rate was 80% (n = 136) with only 74% (n = 126) completing the knowledge portion of the survey. All 10 participants who left the knowledge test blank worked on basic ambulances. The median age of participants was 49 with an interquartile range of 36–54. Table 1 describes the characteristics of the study participants. While none of the participants had attended pediatric-specific CMEs over the past 5 years, 20% reported having had post-graduate pediatric education. A majority of respondents (79.4%) estimated the proportion of their pediatric calls to be consistent with the range documented in the literature and reported by the EMS director for 2012 [6,7]. Thirty percent of respondents estimated that the proportion of pediatric patients transported to hospitals was less than 10% (Table 2).

**Table 1:** Participant characteristics

	**Frequency (%)**	
	**All (n = 136)**	**Did not complete knowledge test (n = 10)**
Sex		
Males	66 (48.5%)	5 (50%)
Females	67 (49.3%)	5 (50%)
No response	3 (2.2%)	-
Age: 22–34	33 (24.3%)	3 (30%)
35–50	43 (31.6%)	3 (30%)
51–65	56 (41.2%)	2 (20%)
66–75	3 (2.2%)	2 (20%)
No response	1 (0.7%)	-
Medical school graduation year		
1968–1984	51 (37.5%)	4 (40%)
1985–1999	46 (33.8%)	3 (30%)
2000–2012	33 (24.3%)	2 (20%)
No response	6 (4.4%)	1 (10%)
Country of medical school		
Armenia	131 (96.3%)	10 (100%)
Azerbaijan	2 (1.5%)	-
Russia	3 (2.2%)	-
No response	None	-
Post-graduate pediatric education		
Yes	27 (19.9%)	3 (30%)
No	93 (68.4%)	6 (60%)
No response	16 (11.8)	1 (10%)
Number of years on ambulance		
Less than 5	38 (27.9%)	3 (30%)
5–15 years	28 (20.6%)	-
16–20 years	10 (7.4%)	1 (10%)
More than 20 years	60 (44.1%)	6 (60%)
No response	None	-
Type of ambulance		
General/Basic	108 (79.4%)	10 (100%)
Resuscitation/Critical care	28 (20.6%)	-
No response	None	-
CME in the past 5 years		
Yes	111 (81.6%)	8 (80%)
No	20 (14.7%)	1 (10%)
No response	5 (3.7%)	1 (10%)

**Table 2:** Use of EMS by pediatric patients

	**<10%,**	**11–25%,**	**26–50%,**	**>50%,**	**Not applicable, %**	**No response, %**
	**%**	**%**	**%**	**%**		
What is the percentage of pediatric patients you see when called?	46.3	33.1	2.2	-	5.1	13.2
What percentage of pediatric calls you receive do you transfer to the hospital?	30.1	22.1	22.1	16.9	0.7	8.1

**Figure 2:**
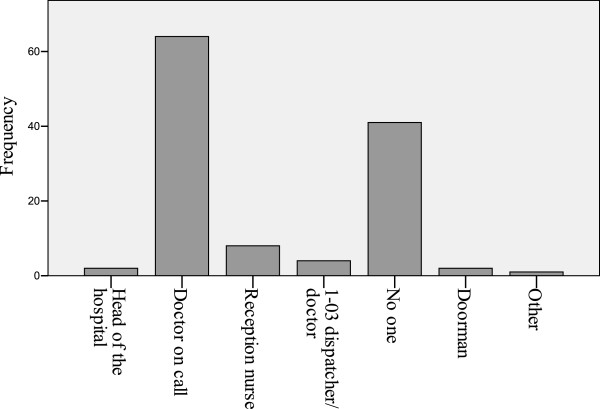
Communications before transition to inpatient care.

The majority of respondents (53%) reported that they have no contact with the on-call physician at the receiving hospital and one-third denied contact with any receiving hospital staff (Figure 2). Respondents identified three pediatric hospitals (Arapgir (42.6%), Mouratsan (50.7%), and St. Mary’s (53%)) most frequently as receiving hospitals. Other hospitals listed included Nork hospital (7.4%) and St. Gregory the Illuminator Emergency Hospital (1.5%). Six percent of providers reported that they would select a receiving hospital based on the patient’s age and specific diagnosis, and 2.2% stated that they would choose the nearest hospital regardless of age and/or diagnosis.

The most common perceived barriers to appropriate PEC were lack of specialized knowledge and skills (38%), lack of pediatric equipment and medications (37%), lack of knowledge and understanding of pediatric medication dosing and pharmacology (23%), and lack of pediatric-specific CME (22%).

**Figure 3:**
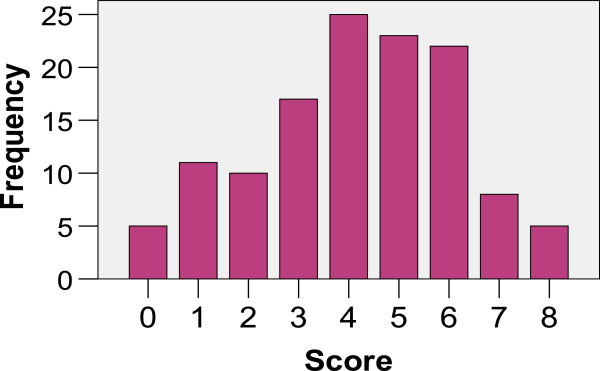
Respondent knowledge score distribution.

The knowledge portion of the survey included 10 true/false/unsure-type questions about PEC (Table 3). The maximum possible score was 10 with a pre-defined passing score of ≥7. The majority (89.7%) failed the test. The mean score was 4.17 ± 1.99 SD (Figure 3). The most frequently incorrect question (59.5%) was regarding the correct dose of epinephrine in anaphylaxis and more than one-third of participants were unsure about the definition of hypotension in children. However, the majority (69.8%) recognized that the compression-to-ventilation ratio varies from neonates to older children. There was no difference in mean score between those who worked on the general/basic ambulance and those who worked on the resuscitation/critical care ambulance (*P* = 0.69; Students *t*-test). The Knowledge Score and passing the test were not associated with variables such as age, post-graduate education, and length of EMS work experience (Tables 4 and 5).

**Table 3:** Knowledge of pediatric rapid assessment and resuscitation (n = 126)

**True/false statements**		**Correct response, %**	**Incorrect response, %**	**Unsure, %**	**No response, %**
1	In CPR the compression-to-ventilation ratio varies from neonates to older children (True)	69.8	13.5	13.5	3.2
2	Chest compressions in a neonate should start for a heart rate less than 100 (False)	53.2	25.4	16.7	4.8
3	Chest compressions in children should start for a heart rate less than 60 (True)	35.7	31.7	21.4	11.1
4	Most children’s heart stops because of respiratory issues (True)	41.3	26.2	16.7	15.9
5	The compression-to-ventilation ratio in neonates is 3:1 (True)	40.5	28.6	17.5	13.5
6	Hypotension in a child under 10 years old is defined as SBP less than 70 + (2 x age) (True)	32.5	15.1	35.7	16.7
7	For 2-person CPR in children, the compression-to-ventilation ratio is 15:2 (True)	50.8	19.8	19.8	9.5
8	For 1-person CPR in children, the compression-to-ventilation ratio is 30:2 (True)	54.8	17.5	17.5	10.3
9	For anaphylaxis, the first medication to give is epinephrine IM 1 mg/mL (1:1,000) at a dose of 0.1 mg/kg (False)	7.1	59.5	21.4	11.9
10	In children, tachycardia can be the only sign of shock (True)	31	31	25.4	12.7

**Table 4:** Relationship between knowledge score and select participant characteristics

**Characteristic**	***P*** **value**
Post-graduate pediatric education	0.35^a^
CME sessions in past 5 years	0.88^a^
Length of time since graduation	0.21^b^
Length of EMS experience	0.10^b^

^a^Students *t*-test, ^b^One-way ANOVA.

**Table 5:** Relationship between passing the test and select participant characteristics

**Characteristic**	***P*** **value**
Age	0.35^a^
Length of EMS experience	0.88^a^
Type of ambulance	0.21^b^
CME attendance	0.10^b^
Post-graduate pediatric training	0.236^c^
Graduation year	0.194^c^
Country of graduation	0.867^c^

^a^Students *t*-test, ^b^One-way ANOVA, ^c^χ^2^ test of independence.

With regard to attitudes, a majority of participants (98.5%) agreed that pre-hospital PEC-related education in Armenia needs to be improved; that there is a need for pediatric-specific CME (98%); and that national out-of-hospital PEC guidelines would increase PEC safety, efficiency, and effectiveness (96%) (Table 6). Seventy percent of respondents agreed with the statement that “there is a lack of standardization regarding pre-hospital PEC in Armenia,” however, only 27.9% felt that this lack of standardization affected their provision of care (Table 7). Univariate analysis using the χ^2^ test of independence did not show a statistically significant difference in attitudes regarding the effect of the absence of standardized care based on physician ambulance type (general/basic ambulance vs. resuscitation/critical care; *P* = 0.95), age (*P* = 0.53), post-graduate pediatric education (*P* = 0.96), or years of experience (*P* = 0.24).

**Table 6:** Attitudes regarding PEC education and efficiency of the emergency system (n = 136)

	**Strongly agree, %**	**Agree, %**	**Disagree, %**	**Strongly disagree, %**	**No response, %**
Pre-hospital care in Armenia is very efficient	16.2	60.3	12.5	4.4	6.6
Pre-hospital PEC related education in Armenia needs improvement	35.3	63.2	1.5	0	0
There is a need for pediatric-specific CME for pre-hospital PEC providers in Armenia	36	62.5	0.7	0	0.7
Pre-hospital PEC guidelines make PEC safer and more efficient and effective	34.6	61.8	1.5	1.5	0.7

**Table 7:** Attitudes regarding standardization of care (n = 136)

	**Yes, %**	**No, %**	**No response, %**	**Not applicable, %**
Is there lack of standardization regarding pre-hospital PEC in Armenia?	70.6	25.7	3.7	n/a
If there is lack of standardization, does it affect the care you provide?	27.9	42.6	17.6	11.8

## Discussion

Children often represent a challenge to emergency and trauma providers because of their behavioral, developmental, anatomic, and physiologic variation compared to adults. In the United States, a study conducted in 2011 in a New Jersey trauma center found pediatric pre-hospital care with regard to endotracheal intubation, peripheral intravenous access, and fluid resuscitation to be suboptimal when compared with adults [8]. Studies in Germany, which follows a similar EMS model to Armenia (i.e., “Franco-German”), have shown that EMS providers are less comfortable and competent in managing pediatric emergencies compared to adult emergencies [7,9]. Similarly, a survey regarding knowledge of pediatric resuscitation guidelines in French EMS teams revealed that a majority lacked sufficient knowledge [10].

Our study demonstrates similar knowledge deficits in pediatric-specific emergency assessment and resuscitation among out-of-hospital EPs in Yerevan, Armenia. This is especially concerning given that there is an association between paramedics’ field performance and their performance on cognitive examinations when assessed by a simulated EMS response [11]. Given the generally lower proportion of pediatric patients seen by the EMS providers and the unique characteristics of children as patients, EMS systems need to take special care to ensure competence and maintenance of skills in their providers. One likely intervention would be the design and implementation of a PEC educational curriculum that meets the needs of providers participating in the EMS system of Yerevan, Armenia.

Our study also highlights many of the systemic issues related to out-of-hospital PEC in Yerevan as perceived by providers, along with substantial deficiencies in pediatric EMS services. The absence of contact with the on-call physician at the receiving hospital in the majority of cases is a significant problem that can result in compromised patient care and safety, and presents a critical opportunity for improvement.

Finally, the absence of national PEC practice guidelines is another deficiency highlighted in this study. Researchers in other countries have shown that clinical practice guidelines improve quality of care and reduce costs [12]. Implementation of such guidelines is dependent on multiple factors, including physician characteristics, enforcement, and methods of dissemination [13]. Moreover, for guidelines to have a positive impact on the quality of care, they need to address not only evidence, but also consider the specific needs of a community, resources, cost, and expert opinion [14]. Physicians who participated in this study are cognizant of this deficiency and of the importance of guidelines; this creates a unique opportunity for action towards the design and implementation of practice-based guidelines for PEC in the Yerevan EMS system.

The results of this study indicate that out-of-hospital EPs in Yerevan, Armenia recognize the importance of pediatric training, their personal deficiencies with regard to PEC, and systemic issues and barriers while expressing a clear desire for improvement. As the government of Armenia works to modernize and improve EMS, this study highlights critical deficits and the strong potential for effective strategies to advance PEC in Armenia.

### Limitations

Consistent with all survey studies, data collected in this study was self-reported and subject to recall and selection bias. Certain questions had a non-response rate of up to 25%. Additionally, the survey may have been underpowered to find difference between some sub-groups.

This study also did not address the knowledge or attitudes of nursing staff on ambulances. As an integral part of the pre-hospital medical team, nursing education would play an important role in the future development of the EMS system in Yerevan, Armenia.

## Conclusions

Out-of-hospital emergency physicians in Yerevan, Armenia are deficient in pediatric-specific emergency assessment and resuscitation knowledge and training, but express a clear desire for improvement. There is a need to support additional PEC training and CME within the EMS system in Armenia.

## Abbreviations

CME: Continuing medical education; EMS: Emergency medical services; EP: Emergency Physicians; MOH: Ministry of health; PEC: Pediatric emergency care; USAID: United States Agency for International Development.

## Competing interests

RD was a paid consultant for Abt Associates and USAID on the Healthcare System Strengthening in Armenia (HS-STAR) Project. The authors declare no other competing interests.

## Authors’ contributions

AB, RD, and HS conceived and designed the study. AB undertook recruitment, supervised data collection, managed data, and performed data analysis. AB, AD, HS, and NC interpreted the data and contributed to the manuscript. All authors read and approved the final manuscript.

## References

[B1] Donaldson RI, Inc AA Situation assessment and improvement strategy of emergency care and ambulance services in Armenia Healthcare System Strengthening in Armenia (HS-STAR) Project 2011 Bethesda, MD: USAID-Healthcare System Strengthening in Armenia (HS-STAR) Project

[B2] Hojnoski JA Ciottone GR Aghababian RV International development of emergency medical systems: educational techniques for the future Eur J Emerg Med 1998 7 1 23 27 10406415

[B3] CIA World Factbook, Armeniahttps://www.cia.gov/library/publications/the-world-factbook/geos/am.html

[B4] Population of the Republic of Armenia – Marzes and Yerevan City. Armstat 2011 http://www.armstat.am/file/article/marz_11_8.pdf

[B5] Yerevan Central Dispatch Center Surveillance Data 2012

[B6] Shah MN Cushman JT Davis CO Bazarian JJ Auinger P Friedman B The epidemiology of emergency medical services use by children: an analysis of the National Hospital Ambulatory Medical Care Survey Prehosp Emerg Care 2008 7 3 269 276 10.1080/10903120802100167 18584491 PMC5237581

[B7] Moller JC Ballnus S Kohl M Gopel W Barthel M Kruger U Friedrich HJ Evaluation of the performance of general emergency physicians in pediatric emergencies: obstructive airway diseases, seizures, and trauma Pediatr Emerg Care 2002 7 6 424 428 10.1097/00006565-200212000-00005 12488835

[B8] Bankole S Asuncion A Ross S Aghai Z Nollah L Echols H Da-Silva S First responder performance in pediatric trauma: a comparison with an adult cohort Pediatr Emerg Care 2011 7 4 e166 170 10.1097/PCC.0b013e3181f36f6e 20729789

[B9] Eich C Roessler M Timmermann A Heuer JF Gentkow U Albrecht B Russo SG Out-of-hospital pediatric emergencies Percept Assess Emerg Physicians Der Anaesthesist 2009 7 9 876 883 10.1007/s00101-009-1603-3 19693447

[B10] Goddet N-S Lode N Descatha A Dolveck F Pès P Chabernaud J-L Baer M Fletcher D National evaluation of knowledge and practice of cardiopulmonary resuscitation of children and infants in the field Ann Fr Anesth Reanim 2009 7 11 943 948 10.1016/j.annfar.2009.09.011 19942396

[B11] Studnek JR Fernandez AR Shimberg B Garifo M Correll M The association between emergency medical services field performance assessed by high-fidelity simulation and the cognitive knowledge of practicing paramedics Acad Emerg Med 2011 7 11 1177 1185 10.1111/j.1553-2712.2011.01208.x 22092899

[B12] Woolf SH Grol R Hutchinson A Eccles M Grimshaw J Clinical guidelines: potential benefits, limitations, and harms of clinical guidelines Br Med J 1999 7 7182 527 530 10.1136/bmj.318.7182.527 10024268 PMC1114973

[B13] Dean A Davis DA Taylor-Vaisey A Translating guidelines into practice Can Med Assoc J 1997 7 4 408 416 PMC1227916 9275952

[B14] Grol R Improving the quality of medical care: building bridges among professional pride, payer profit, and patient satisfaction JAMA 2001 7 20 2578 2585 10.1001/jama.286.20.2578 11722272

